# Revision surgery versus biologic treatment with omalizumab in recurrent chronic rhinosinusitis with nasal polyps: An analysis of cost-utility and clinical outcomes^☆^

**DOI:** 10.1016/j.waojou.2023.100846

**Published:** 2023-11-29

**Authors:** Yutong Sima, Jing Zhang, Ming Zheng, Yan Zhao, Xiangdong Wang, Luo Zhang

**Affiliations:** a Department of Otolaryngology Head and Neck Surgery, Beijing Tongren Hospital, Capital Medical University, Beijing 100730, China; b Beijing Laboratory of Allergic Diseases, Beijing Municipal Education Commission and Beijing Key Laboratory of Nasal Diseases, Beijing Institute of Otolaryngology, Beijing 100005, China; c Department of Otolaryngology Head and Neck Surgery, Beijing Anzhen Hospital, Capital Medical University, Beijing 100029, China; d Department of Allergy, Beijing Tongren Hospital, Capital Medical University, Beijing 100730, China; e Research Unit of Diagnosis and Treatment of Chronic Nasal Diseases, Chinese Academy of Medical Sciences, Beijing 100005, China

**Keywords:** Recurrent chronic rhinosinusitis with nasal polyps, Omalizumab, Revision surgery, SNOT-22, Economic cost, Duration cost

## Abstract

**Background:**

Both revision surgery and omalizumab are recommended therapies for the treatment of recurrent chronic rhinosinusitis with nasal polyps (CRSwNP) and can improve patients' clinical symptoms and quality of life (QoL). The aim of this study was to compare the improvement in sinus-related symptoms, QoL, economic cost, and duration cost between treatment with revision-surgery and treatment with omalizumab.

**Methods:**

This was a prospective study of patients with recurrent CRSwNP. All patients were asked to complete a 22-item sino-nasal outcome test (SNOT-22), a visual analog scale (VAS), and a 36-item short-form (SF-36) questionnaire at baseline and 6 months after the treatments. Patients were required to document economic costs and duration costs within 6 months and report them at each visit.

**Results:**

A total of 44 patients who received the treatment of revision surgery or omalizumab were enrolled in this study. After six months of treatment, the improvements in total SNOT-22 and SF-36 in 8 domains were not different between the 2 treatments. The improvements in rhinologic symptoms, extranasal rhinologic symptoms, and ear/facial symptoms according to the SNOT-22 (*P* value = 0.0288, 0.0016, and 0.0347, respectively) and the improvements in nasal congestion, loss of smell, and overall symptoms assessed by the VAS (*P* value = 0.0057, 0.0206, and 0.0122, respectively) were better in the revision surgery group than in the omalizumab group. The economic cost and the total duration cost were obviously lower in the omalizumab group (¥18836 and 1 day) than in the revision surgery group (¥29824 and 23 days).

**Conclusions:**

Both revision surgery and omalizumab treatments can improve the clinical symptoms and QoL of patients with recurrent CRSwNP. Patients who underwent revision surgery experienced better improvement in sinus-related symptoms. However, omalizumab treatment clearly showed a benefit in terms of economic cost and duration cost of disease-related care.

## Introduction

Chronic rhinosinusitis (CRS) is a chronic inflammatory disease of the sinuses and nasal mucosa characterized by nasal congestion, pus flow, loss of smell, and head and face swelling and pain with a duration of more than 12 weeks.^1^ The prevalence of CRS is 10.9% in Europe, and the disease affects 2–8% of the population in China.^2^^,^^3^ CRS seriously affects patients' lives, work, and studies and increases the overall medical burden on patients and society.^1^ CRS is categorized into 2 clinical phenotypes based on the presence or absence of nasal polyps: chronic rhinosinusitis with nasal polyps (CRSwNP) and chronic rhinosinusitis without nasal polyps (CRSsNP).^4^ Depending on the endotype characteristics, CRS is classified as Type 2 (T2) CRS or non-T2 CRS. In Europe, approximately 80% of patients with CRSwNP showed T2 inflammatory features.^5^, ^6^, ^7^ T2 inflammation is also emerging as the main inflammatory endotype of CRSwNP in China.^8^, ^9^, ^10^, ^11^ T2 CRS is characterized by high levels of interleukin (IL)-4, IL-5, IL-13, and IgE and increased eosinophil counts and has a high postoperative recurrence rate, severe clinical symptoms, and comorbidities.^8^^,^^12^, ^13^, ^14^, ^15^ Current medical and surgical treatments, such as local or systemic glucocorticoids, endoscopic sinus surgery, and monoclonal antibodies targeting T2 biomarkers (including dupilumab, omalizumab, and mepolizumab), may be associated with the recurrence of nasal polyps.^16^ Approximately 25–30% of patients with T2 inflammatory relapse even received oral glucocorticoids or conventional sinus surgery.^17^ With the development of medicine, monoclonal antibodies have achieved clinical efficacy in the treatment of patients with nasal polyps.^18^ Each treatment has limitations and advantages.^19^ For endoscopic sinus surgery (ESS) treatment in patients with CRS, it is efficacious for either phenotypes or endotypes. However, the potential negative effects of anesthesia and lack of anatomical clarity due to second or multiple surgeries in ESS cannot be ignored.^20^^,^^21^ Some patients with CRSwNP prefer nonsurgical interventions for treatment.^22^ Biologics have shown positive results in reducing nasal polyp size, alleviating nasal congestion, improving patient-reported outcomes, and decreasing the need for surgery and systemic corticosteroids.^23^, ^24^, ^25^ Biologic effectiveness is dependent on the continued application of monoclonal antibodies and specific populations. For patients with uncontrolled CRSwNP, biologics and revision surgery can be candidate treatment options. However, there is still no clear judgment on which treatment can provide greater benefit to patients. In addition to the improvement in clinical symptoms, the economic cost and duration cost are also important factors that affect the choice of treatment.^21^^,^^26^ Omalizumab is a widely used therapeutic monoclonal antibody targeting free IgE. The United States (US) Food and Drug Administration (FDA) approved omalizumab for the treatment of severe CRSwNP.^7^ Omalizumab has also demonstrated efficacy and safety in Chinese patients with refractory CRSwNP.^27^ Therefore, we evaluated the difference in improvement of sinus-related clinical symptoms and general quality of life, economic cost and duration cost in patients with recurrent CRSwNP who received revision surgery or omalizumab treatment. We hope the results can contribute to clinical treatment management and provide better options for patients.

## Materials and methods

### Patients

This is a prospective study of recurrent CRSwNP patients conducted from March 1, 2020, to December 31, 2022. According to the guidelines of European Position Paper on Rhinosinusitis and Nasal Polyps 2020 (EPOS2020)^1^ and the European Forum for Research and Education in Allergy and Airway disease (EUFOREA) expert consensus,^28^ patients can be treated with revision surgery or monoclonal antibodies. Patients will make their own choice according to the advantages and disadvantages of each treatment. To minimize the bias of the study, patients who received revision surgery treatment were treated by the same senior rhinology specialist. The patients received regular postoperative medication and regular outpatient visits based on sinus recovery. Omalizumab treatment was administered using standard dosing according to the body weight (kilogram) and the pretreatment serum total IgE level (kU/L). Patients who were younger than 18 years old or older than 70 years old or who had fungal rhinosinusitis or autoimmune diseases or who had ever received monoclonal antibody treatment or undergone more than 2 surgical procedures for nasal polyps were excluded from the study.

The Ethics Committee of Beijing Tongren Hospital and Chinese Clinical Trial Registry authorized this study, and all patients supplied written informed consent.

### Assessments

Patients’ demographic data, including sex, age, body mass index (BMI), IgE level, region of residence, education attainment, monthly salary, smoking status, and comorbidities, were collected before the treatment. We asked all patients to document economic costs and duration costs within 6 months. The current economic cost and duration cost at each visit were reported. The economic cost included the disease-related cost and the transportation cost for medical care. The duration cost included the outpatient visit time, hospitalization time (only for revision surgery patients), and postoperative time (only for revision surgery patients). Some patients come from other cities, which increased indirect economic costs, including the cost of food and accommodation, caused a large bias. Therefore, we only analyzed the data from the same city.

Patients were asked to report the clinical symptoms of nasal obstruction, runny nose, facial pain, headache, sense of smell, and overall symptoms on the CRS disease severity by a visual analog scale (VAS) (score range, 0–10, from not troublesome to worst thinkable troublesome) at baseline and 24 weeks after treatment. The Sino-Nasal Outcome Test-22 (SNOT-22) questionnaire (total score range, 0–110) at baseline and 24 weeks was documented to assess the symptoms, sleep, functional, and emotional consequences of CRS; in this questionnarie, patients responded to 22 items by using a 6-category range (0–5, from no problems to problems as bad as it can be). The 22 items of the SNOT-22 questionnaire were categorized and summarized into 5 separate domains with minimal item cross-loading: rhinologic symptoms (score range, 0–30), which included items 1, 2, 3, 4, 5, and 8; extranasal rhinologic symptoms (score range, 0–15), which included items 6, 7, and 8; ear/facial symptoms (score range, 0–25), which included items 2, 9, 10, 11, and 12; psychological dysfunction (score range, 0–35), which included items 16, 17, 18, 19, 20, 21, and 22; and sleep dysfunction (score range, 0–25), which included items 13, 14, 15, 16, and 17.^29^

The 36-item short-form (SF-36) health survey is a general health-related quality of life (HRQoL) questionnaire that is used to evaluate health status using a 36-item questionnaire. It measures 8 different aspects of health, including physical functioning (PF), role-physical (RP), bodily pain (BP), general health (GH), vitality (VT), social functioning (SF), role-emotional (RE), and mental health (MH).

### Statistical analysis

GraphPad Prism version 9 (GraphPad Software Inc., San Diego, CA) was used to analyze this study. Baseline characteristics were presented as the mean ± SD or median and interquartile range (IQR) for continuous variables and the number (%) for categorical variables. The Kolmogorov‒Smirnov test was used to analyze the normality of the data. Fisher's test was conducted to compare sex, region of residence, education attainment, smoking status, comorbidity, and salary monthly between the 2 groups. The Mann‒Whitney test was used to compare age, BMI, IgE level, and mean change of clinical symptom scores between the two groups. A *P* < 0.05 (two-tailed) was set as statistically significant.

## Results

### Demographic and baseline clinical characteristics

Forty-four patients with refractory CRSwNP were enrolled in this prospective study. Twenty-two individuals chose revision surgery treatment, and 22 individuals chose omalizumab treatment. All patients completed six-month visits and related requirements.

There were no significant differences in baseline characteristics, including sex, age, BMI, IgE, region of residence, education attainment, smoking status, comorbidity, and salary monthly, between the 2 groups (all *P* values > 0.05) (Table 1). For the clinical symptoms at baseline for the 2 groups, the total SNOT-22 score and 5 separate domains of SNOT-22 and SF-36 score in 8 domains did not show any significant difference (all *P* values > 0.05). More severity in nasal congestion by VAS was exhibited in the revision surgery group (IQR: 7.00–9.50) than in the omalizumab group (IQR: 6.48–8.48) (*P* = 0.0257). Other symptoms of runny nose, facial pain, headache, loss of smell, and overall symptoms as assessed by VAS showed no significant differences (all *P* values > 0.05) (Table 2).

**Table 1:** The demographic and clinical characteristics of participants

Characteristics	Revision surgery (N = 22)	Omalizumab (N = 22)	*P* value
Sex			
Male, n (%)	11 (50.00)	11 (50.00)	NS
Female, n (%)	11 (50.00)	11 (50.00)	
Age (years), mean ± SD	44.18 ± 10.77	46.50 ± 12.11	0.5566
BMI (kg/m^2^), median (IQR)	23.75 (21.58–26.05)	23.75 (21.80–27.45)	0.9769
IgE (kU/L), median (IQR)	127.00 (58.43–317.80)	167.50 (70.58–374.80)	0.5677
Region of residence			
Urban, n (%)	21 (95.45)	21 (95.45)	NS
Rural, n (%)	1 (4.55)	1 (4.55)	
Education attainment			
College and higher, n (%)	15 (68.18)	17 (77.27)	0.4984
Others, n (%)	7 (31.2)	5 (22.73)	
Smoking status			
Never, n (%)	16 (72.73)	15 (68.18)	0.6748
Previous, n (%)	4 (18.18)	3 (13.64)	
Current, n (%)	2 (9.09)	4 (18.18)	
Comorbidity			
Allergic rhinitis, n (%)	20 (90.91)	22 (100.00)	0.4035
Asthma, n (%)	8 (36.36)	22 (100.00)	
Atopic dermatitis, n (%)	4 (18.18)	6 (27.27)	
AERD, n (%)	1 (4.55)	0 (0.00)	
Otitis media, n (%)	4 (18.18)	4 (18.18)	
Allergic conjunctivitis, n (%)	1 (4.55)	2 (9.09)	
Salary monthly			
less than 5000, n (%)	6	2	0.2668
5000–10000, n (%)	3	5	
more than 10000, n (%)	13	15	
Current, n (%)	2 (9.09)	4 (18.18)

**Table 2:** The clinical symptoms of participants at baseline

Characteristics	Revision surgery (N = 22)	Omalizumab (N = 22)	*P* value
SNOT-22 (scale 0–110), median (IQR)	38.75 (38.75–79.00)	47.00 (26.00–64.50)	0.1386
Rhinologic symptoms (scale 0–30), median (IQR)	19.50 (16.00–23.25)	16.50 (10.75–22.50)	0.2128
Extranasal rhinologic symptoms (scale 0–15), median (IQR)	13.00 (10.00–14.00)	11.00 (5.75–13.50)	0.1555
Ear/facial symptoms (scale 0–25), median (IQR)	10.50 (4.50–12.25)	7.00 (2.00–11.25)	0.1755
Psychological dysfunction (scale 0–35), median (IQR)	15.00 (7.00–28.75)	11.00 (5.50–20.25)	0.3600
Sleep dysfunction (scale 0–25), median (IQR)	10.50 (7.75–21.25)	8.00 (4.00–12.50)	0.0670
Visual analog scale, VAS (scale 0–10)			
Nasal obstruction, median (IQR)	8.50 (7.00–9.50)	7.00 (6.48–8.48)	**0.0257∗**
Runny nose, median (IQR)	7.75 (4.75–9.00)	6.35 (4.00–8.25)	0.3600
Facial pain, median (IQR)	1.00 (0.20–5.00)	1.60 (0.35–4.10)	0.8385
Headache, median (IQR)	2.25 (1.00–5.38)	2.70 (0.50–5.53)	0.9120
Sense of smell, median (IQR)	9.00 (7.25–9.73)	9.20 (5.18–10.00)	0.7381
Overall symptoms, median (IQR)	7.75 (5.38–9.00)	6.85 (4.00–7.15)	0.0519
36-item short-form health survey (SF-36)			
Physical functioning, median (IQR)	80.00 (50.00–96.25)	92.50 (75.00–95.00)	0.2390
Role-physical, median (IQR)	62.50 (0.00–100.00)	75.00 (21.25–100.00)	0.4362
Bodily pain, median (IQR)	74.00 (52.00–100.00)	82.00 (74.00–100.00)0.0	0.0771
General health, median (IQR)	52.50 (30.00–77.75)	47.50 (35.00–67.00)	0.7754
Vitality, median (IQR)	67.50 (50.00–76.25)	62.50 (50.00–70.00)	0.4848
Social functioning, median (IQR)	75.00 (47.00–91.00)	81.25 (71.88–88.00)	0.6758
Role-emotional, median (IQR)	67.00 (0.00–100.00)	100.00 (24.75–100.00)	0.2290
Mental health, median (IQR)	68.00 (56.00–86.00)	64.00 (52.00–77.00)	0.4656

∗, *P* < 0.05. IQR, interquartile range.

### The economic cost in revision surgery patients and omalizumab treatment patients

We summarized the direct economic cost (disease-related cost) and indirect economic cost (transportation cost) of 2 methods of treatment for patients with recurrent CRSwNP. The total costs of disease-related complications were obviously lower in the omalizumab group (IQR: ¥13517–23323) than in the revision surgery group (IQR: ¥27824–33318) (*P* < 0.0001). The cost of transportation showed no significant difference between the 2 groups (Table 3).

**Table 3:** The economic difference between patients with revision surgery and omalizumab treatment

Characteristics	Revision surgery (N = 22)	Omalizumab (N = 22)	*P* value
Total cost of disease-related (¥), median (IQR)	29824 (27824–33318)	18836 (13517–23323)	**< 0.0001∗**
Total cost of transportation (¥), median (IQR)	32.50 (10.00–500.00)	30.00 (10.00–100.00)	0.6115

∗, *P* < 0.05. IQR, interquartile range

### The duration cost in revision surgery patients and omalizumab treatment patients

For patients undergoing revision surgery treatment, data regarding the duration of hospitalization for surgery and recovery after surgery was required in addition to information about duration of outpatient visits. Patients who chose revision surgery treatment (IQR: 9.00–78.00) had longer total durations of outpatient visits than patients who received omalizumab treatment (IQR: 12.00–28.50) (*P* < 0.0001), and the total duration of disease-related visits (IQR: 509.00–600.00) was obviously higher than that in the omalizumab group (*P* < 0.0001) (Table 4).

**Table 4:** The duration of sick leave difference between patients with revision surgery and omalizumab treatment

Characteristics	Revision surgery (N = 22)	Omalizumab (N = 22)	*P* value
Total duration of outpatient visits (hours), median (IQR)	24.00 (9.00–78.00)	24.00 (12.00–28.50)	0.9488
Total duration of hospitalization (hours), median (IQR)	168.00 (138.00–192.00)	/	/
Total duration of postoperative (hours), median (IQR)	336.00 (336.00–336.00)	/	/
Total duration of disease-related (hours), median (IQR)	547.50 (509.00–600.00)	24.00 (12.00–28.50)	**< 0.0001∗**

∗, *P* < 0.05. IQR, interquartile range. The data was the total duration in six-month follow-up period

### Clinical symptom changes in revision surgery patients and omalizumab treatment patients

**Fig. 1:**
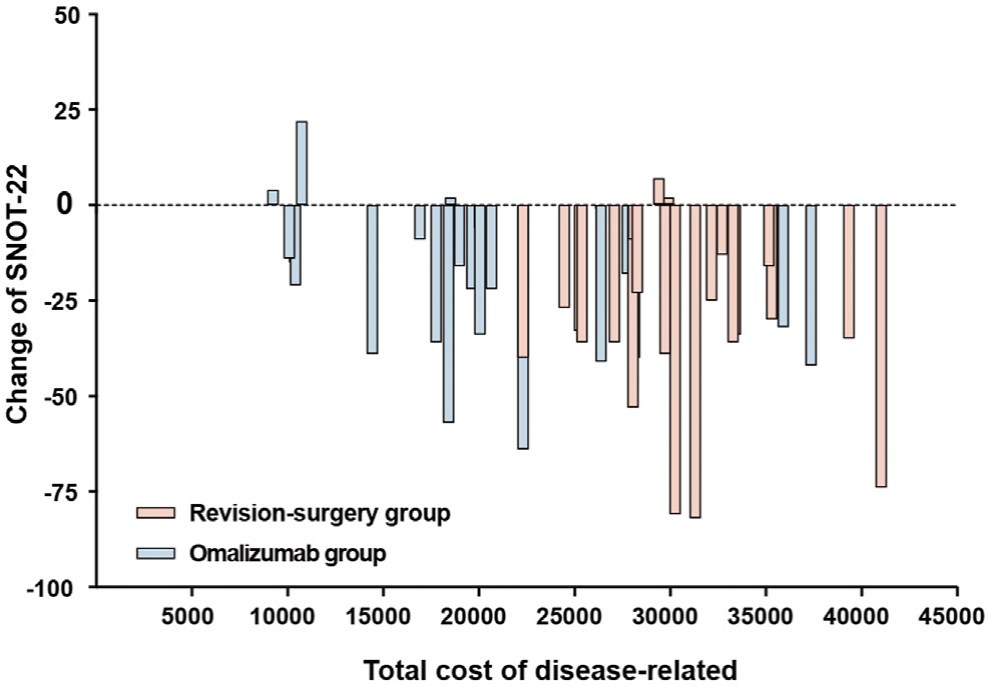
The economic cost and the SNOT-22 improvement for all participants Pink indicates patients who received revision surgery treatment, and blue indicates patients who received omalizumab treatment. SNOT-22, 22-item sino-nasal outcome test

**Fig. 2:**
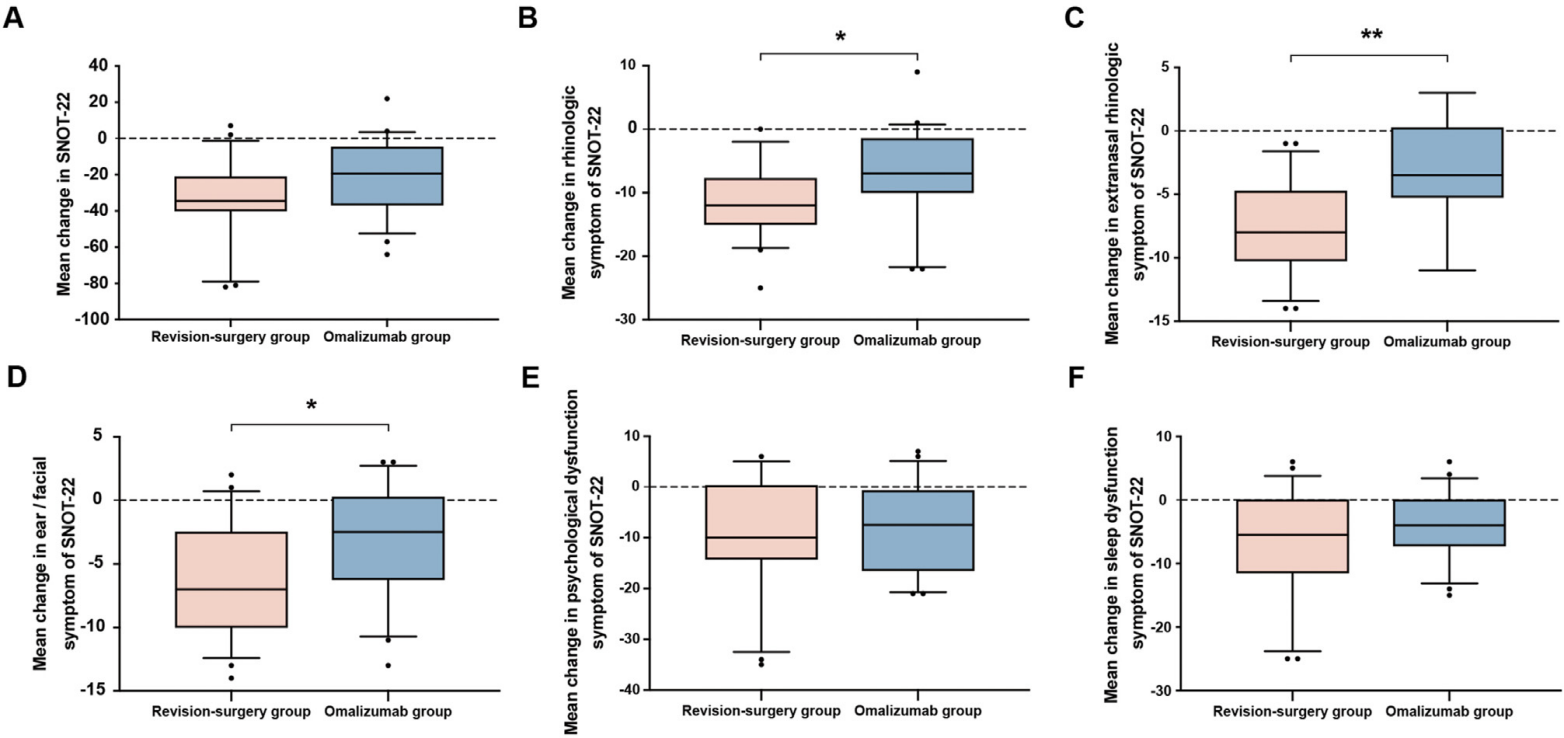
The mean change in (**A**) the total SNOT-22 and (**B–F**) the five separated domains of SNOT-22 in the two groups. The error bar is the median (10th and 90th percentile). SNOT-22, 22-item sino-nasal outcome test; ∗, *P* < 0.05; ∗∗, *P* < 0.01

The economic cost and the SNOT-22 improvement for all participants are shown in Fig. 1. Patients’ SNOT-22 assessments were improved with either of the two treatments after six months. We compared the improvement in patients' symptoms at baseline and after six months of treatment in both groups of patients. The total SNOT-22 improvement based on sinus disease did not show a difference between the 2 groups (Fig. 2A). During further analysis based on the SNOT-22, we found that the improvements in rhinologic symptoms (median −12.00 vs. −7.00) (Fig. 2B), extranasal rhinologic symptoms (median −8.00 vs −3.50) (Fig. 2C), and ear/facial symptoms (median −7.00 vs−2.50) (Fig. 2D) in the revision surgery group were more obvious than those in the omalizumab group (*P* value = 0.0288, 0.0016, and 0.0347, respectively). The improvements in psychological dysfunction symptoms (Fig. 2E) and sleep dysfunction symptoms (Fig. 2F) of SNOT-22 were not different between the 2 groups.

**Fig. 3:**
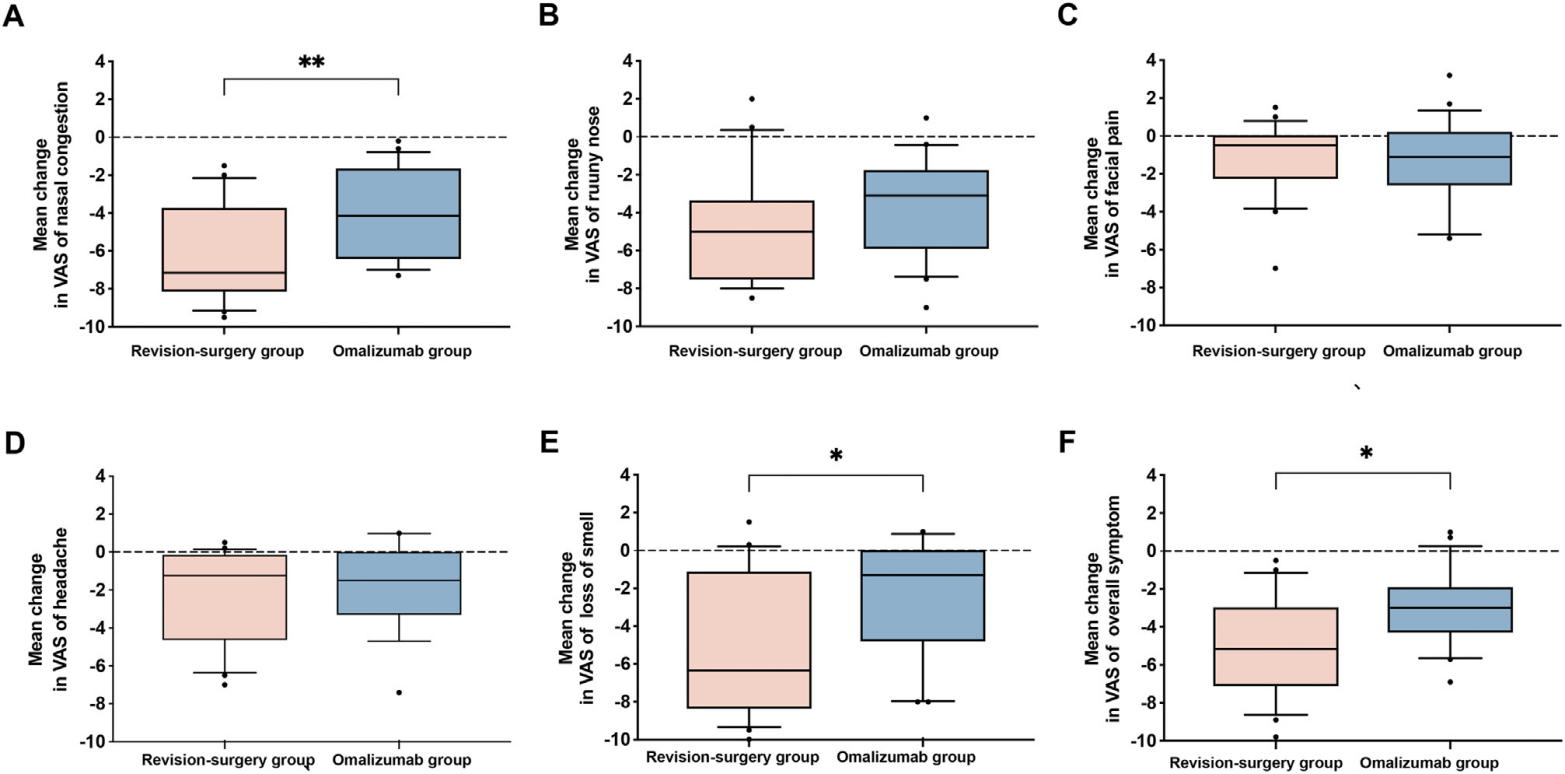
The mean change in (**A**) nasal congestion, (**B**) runny nose, (**C**) facial pain, (**D**) headache, (**E**) loss of smell, and (**F**) total symptoms in the two groups. The error bar is the median (10th and 90th percentile). VAS, visual analog scale; ∗, *P* < 0.05; ∗∗, *P* < 0.01

**Fig. 4:**
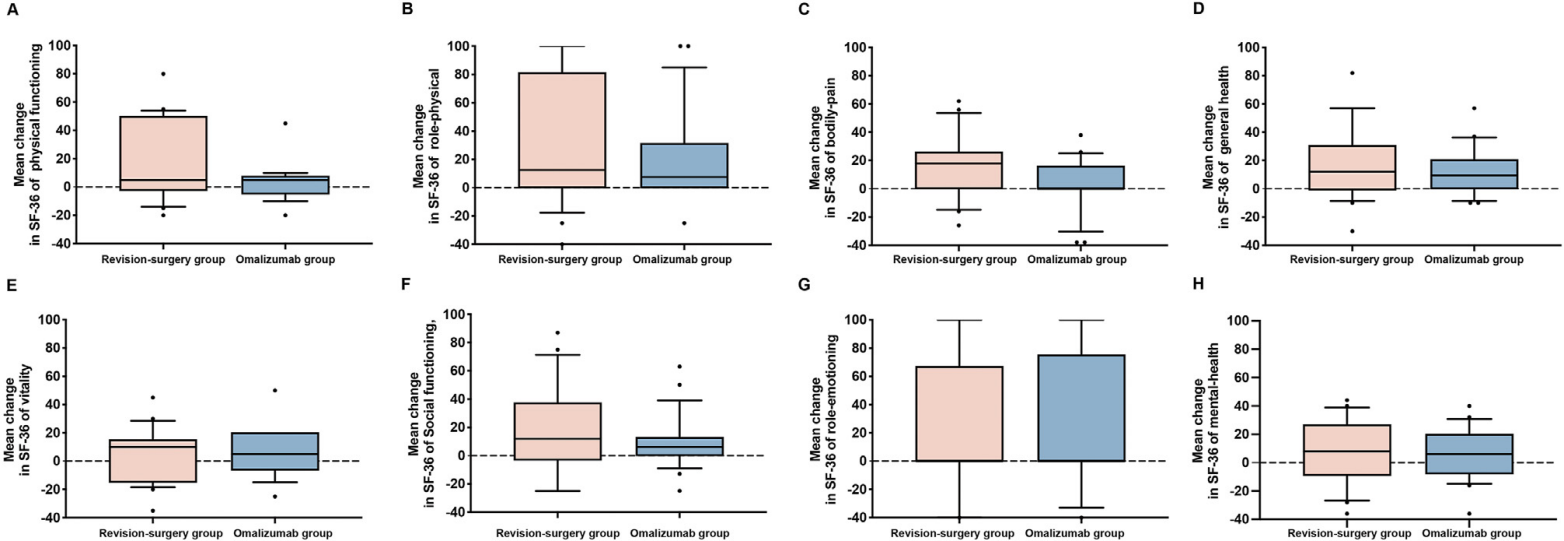
The mean change in the SF-36 in (**A-H**) 8 domains in the two groups The error bar is the median (10th and 90th percentile). SF-36, 36-item short-form

The levels of nasal congestion (median −7.15 vs −4.15) (Fig. 3A), loss of smell (median −6.35 vs −1.30) (Fig. 3E), and overall symptom (median −5.15 vs −3.00) (Fig. 3F) status by VAS in the revision surgery group showed more obvious improvements than those in the omalizumab group (*P* value = 0.0057, 0.0206, and 0.0122, respectively). Patients' improvements in runny nose (Fig. 3B), facial pain (Fig. 3C), and headache (Fig. 3D) by VAS were not different between the 2 groups. In patients’ generalized health improvement based on SF-36, the 8 domains, including PF, RP, BP, GH, VT, SF, RE, and MH, were not different between the revision surgery group and the omalizumab treatment group (Fig. 4).

## Discussion

Currently, the preferred treatment for patients with CRSwNP is nasal glucocorticoids combined with doxycycline for 3 months and eventually oral glucocorticoids for patients with uncontrolled symptoms. Surgery is always a possibility after recommended medical therapy has failed. The primary objectives of ESS are to clear blocked sinus outflow, remove inflammatory tissue, and offer the greatest possible access to topical medications. For patients with recurrent CRSwNP, ESS can improve patients’ disease-related clinical symptoms, even for patients comorbid with asthma.^30^

Biologics are also a recommended treatment option for patients who have failed conservative medications or who have relapsed after surgery, especially for type 2 endotype patients. Almost in all regions, IgE was elevated in type 2 inflammation, and which is thought to be an important mediator of type 2 inflammation.^5^ Omalizumab is a humanized anti-IgE monoclonal antibody (mAb). Omalizumab decreases the quality of free IgE that can bind to the Fc ε RI receptor on effector cells.^31^ Consequently, antigen-bound IgE cannot cross-link with receptors, blocking the production of proinflammatory cytokines and improves patients’ clinical symptoms. Both treatments can be beneficial for patients with CRSwNP, while the recurrence and risk of surgery and the efficacy and safety of long-term biologic therapy still need to be further evaluated.

The SNOT-22 is the most extensively and popularly used disease-specific questionnaire and has been divided into 5 subdomains examining concerns related to rhinologic, extrarhinologic, ear and facial, psychological, and sleep-induced disorders.^32^ Previous studies have confirmed that both surgical and biological treatments can improve patients' SNOT-22 total scores.^27^^,^^33^ In our study, we compared 2 treatments for recurrent CRSwNP, and the improvement in total SNOT-22 did not show a difference (*P* = 0.0751). The median improvements in revision surgery and omalizumab treatment were −34.50 vs. −19.50, respectively. We further analyzed the improvement of the two treatments in 5 domains. Patients treated with revision surgery can obtain greater benefit in rhinologic symptoms, extranasal rhinologic symptoms, and ear/facial symptoms after 6 months compared with omalizumab-treated patients. The VAS results can directly depict the patient's clinical symptoms. We found that patients who chose revision surgery therapy had more severe symptoms of nasal congestion at baseline than patients who chose omalizumab therapy (median 8.50 vs median 7.00). Nasal congestion may be an important consideration for patients making treatment decisions. The improvements in nasal congestion (median −7.15 vs median −4.15), loss of smell (median −6.35 vs median −1.30), and overall symptoms (median −5.15 vs median −3.00) in revision surgery were better than those in the omalizumab group. It is easy to understand that surgery removes inflammatory tissue and nasal polyps, which can thoroughly improve the severity of nasal-related and extranasal-related sinus symptoms. Loss of smell in CRS is caused by multifactorial combinations. Relieving mechanical restriction (including edema or nasal polyps) of odorant transmission in the olfactory cleft can solve the problem of loss of smell.^34^^,^^35^ In patients with CRSwNP, nasal obstruction and loss of smell have been found to be clinical markers of disease severity. Surgery can resolve mechanical obstruction, improve the olfactory epithelium, and facilitate recovery of olfactory function in CRS patients.^35^^,^^36^ Omalizumab treatment significantly improves patients' sense of smell, and a sustained effect is still observed with long-term treatment. However, at the end of omalizumab treatment, patients' sense of smell is diminished.^37^ The discontinuation of biologic therapy in patients with type 2 CRSwNP will result in a rebound of nasal symptoms, and continued treatment with monoclonal antibodies is necessary to maintain clinical improvement in patients with CRSwNP.^38^ Importantly, both revision surgery and omalizumab therapy are beneficial for patients with recurrent CRSwNP.^27^^,^^30^^,^^39^ Although the improvement in loss of smell in the revision surgery group was better than that in the omalizumab group after 6 months of clinical observation, long-term follow-up of the effect of surgery on olfactory function remains to be considered.^35^^,^^36^

Health-related quality of life (HRQoL) is an important part of clinical management. Patients with CRSwNP have significantly lower HRQoL,^40^ and patients' disease severity, comorbidities, and refractory disease are positively correlated with the degree of impact on HRQoL.^41^ The SF-36 health survey is a popular questionnaire that fulfills stringent criteria of reliability and validity.^42^ Patients with CRSwNP had lower overall scores, and the impact of several aspects of CRSwNP in SF-36 was even greater than angina or chronic respiratory disease.^43^ Surgery treatment and omalizumab therapy both improve patients’ quality of life, which was measured by the SF-36 questionnaire.^25^^,^^44^ In our study, we found that there was no difference in the improvement in quality of life in patients with recurrent CRSwNP between the revision surgery group and the omalizumab group.

CRSwNP has considerable direct and indirect costs to patients and society. The direct and indirect costs for CRSwNP patients in Europe are €1501/person/year and €5659/person/year, respectively.^45^^,^^46^ In the United States, annual healthcare costs for CRSwNP patients totaled $11 507, and the average CRS patient had 4.8 days of absenteeism per year.^47^^,^^48^ Economic cost is also an important consideration for patients and ear, nose, and throat (ENT) physicians when making clinical choices. For patients undergoing surgical treatment, in addition to the cost of the surgery, there is the cost of postsurgical follow-up treatment. In our study, we compared the actual economic cost in two different treatments for recurrent CRSwNP. The cost including the surgery or omalizumab, and all medicine related to nasal disease, such as intranasal corticosteroid, antihistamine, or anti-leukotriene. The median cost in patients undergoing revision surgery was approximately 1.6 times of patients treated with omalizumab (¥29824 vs. ¥18836). CRS-related economic costs of both methods of treatment are clearly lower than those in Europe and the United States. Both dupilumab and omalizumab are monoclonal antibodies against type 2 CRS. However, the two monoclonal antibodies that differ in the frequency of treatment and product value. One study compared the cost-effectiveness of ESS and dupilumab in patients with CRSwNP and found that dupilumab treatment was more costly and less effectively than ESS. And no matter the frequency of revision surgery, the yearly cost of dupilumab treatment was $855 higher than ESS treatment.^18^ This is a very practical study for real-world clinical management.

The duration cost is another important factor in the consideration of treatment options.

The duration cost of omalizumab therapy is related only to the outpatient visit, while the duration cost in revision surgery patients includes hospitalization, missed work during the postoperative recovery, and postoperative outpatient follow-up (the total duration cost: median 1 day vs 23 days). Therefore, our study demonstrates the absolute advantage of omalizumab in terms of disease-related economic cost and duration cost. We found that the duration of recovery after surgery is long, which may reflect the trauma to the body caused by anesthesia and surgery.

Given its obvious value, our study also has some shortcomings. First, we only counted the economic cost and duration costs of patients, without accounting for the related costs of accompanying patients. In addition, this study only considered quantifiable costs, and the impact of working with illness on productivity and the impact of absenteeism on employer welfare were not included. Third, the number of patients included in this study was small, and further research is still needed to further expand the range of patients and hospitals at different economic levels.

## Conclusion

CRSwNP is an inflammation-mediated disease with an under-recognized clinical, humanistic, social, economic, and duration burden. A high recurrence rate of CRSwNP after existing medical and surgical interventions worsens the stress experienced by patients and ENT surgeons. The advantages and disadvantages of treatments need to be fully considered in the clinical decision. In summary, this study compared the clinical outcomes and cost utility of omalizumab treatment and revision surgery treatment in patients with recurrent CRSwNP. It is hoped that the findings will be valuable for the clinical management of patients with recurrent CRSwNP.

## Abbreviations

CRS, chronic rhinosinusitis; CRSwNP, chronic rhinosinusitis with nasal polyp; SNOT-22, 22-item sino-nasal outcome test; VAS, visual analog scale; SF-36, 36-item short-form; QoL, quality of life; T2, Type 2

## Funding

This project was supported by the National Key R&D Program of China (2022YFC2504100), the Program for the Changjiang Scholars and Innovative Research Team (IRT13082), the 10.13039/100014717National Natural Science Foundation of China (82171110, 82000962, and 81970852), the CAMS Innovation Fund for Medical Sciences (2019-I2M-5–022), the Capital's funds for health improvement and research (2022-1-1091), the Beijing Natural Science Foundation (7222024), the Beijing New-star Plan of Science and Technology (20220484226), the Beijing Hospitals Authority Youth Program (QML20230201), the Public Welfare Development and Reform Pilot Project (2019-10), the Beijing Municipal Science & Technology Commission (Z211100002921057) and the Special Funds for the Construction of High-level Public Health Technical Talents (Lingjunrencai-01-08 and Lingjunrencai-02-09).

## Availability of data and material

The corresponding author will provide the datasets that support the study's findings on reasonable request.

## Author contribution

YS, JZ, MZ, YZ, XW, and LZ conceived and collected the clinical data. YS and JZ drafted the manuscript. LZ and XW supervised and conceptualized the study and were involved in the important revision of the manuscript. YS and JZ performed the data analysis. All the authors have read and approved the final version of the manuscript.

## Ethics approval

This study was approved by the Ethics Committee of Beijing Tongren Hospital and Chinese Clinical Trail Registry. And written informed consent was obtained from all participants.

## Consent for publication

All authors have read the final manuscript and agreed to publication of the work.

## Declaration of competing interest

All the authors declare that they have no conflicts of interest related to the contents of this work.
